# Magnetic moment evolution and spin freezing in doped BaFe_2_As_2_

**DOI:** 10.1038/s41598-017-07286-6

**Published:** 2017-08-14

**Authors:** Jonathan Pelliciari, Yaobo Huang, Kenji Ishii, Chenglin Zhang, Pengcheng Dai, Gen Fu Chen, Lingyi Xing, Xiancheng Wang, Changqing Jin, Hong Ding, Philipp Werner, Thorsten Schmitt

**Affiliations:** 1 Research Department Synchrotron Radiation and Nanotechnology, Paul Scherrer Institut, CH-5232 Villigen PSI, Switzerland; 2 Beijing National Lab for Condensed Matter Physics, Institute of Physics, Chinese Academy of Sciences, Beijing, 100190 China; 3 Synchrotron Radiation Research Center, National Institutes for Quantum and Radiological Science and Technology, Sayo, Hyogo 679-5148 Japan; 4 Department of Physics and Astronomy, Rice University, Houston, Texas 77005 USA; 5 Collaborative Innovation Center for Quantum Matters, Beijing, China; 6 Department of Physics, University of Fribourg, Chemin du Musée 3, CH-1700, Fribourg, Switzerland; 7 Department of Physics, Massachusetts Institute of Technology, Cambridge, MA 02139 USA

## Abstract

Fe-K_*β*_ X-ray emission spectroscopy measurements reveal an asymmetric doping dependence of the magnetic moments *μ*_bare_ in electron- and hole-doped BaFe_2_As_2_. At low temperature, *μ*_bare_ is nearly constant in hole-doped samples, whereas it decreases upon electron doping. Increasing temperature substantially enhances *μ*_bare_ in the hole-doped region, which is naturally explained by the theoretically predicted crossover into a spin-frozen state. Our measurements demonstrate the importance of Hund’s-coupling and electronic correlations, especially for hole-doped BaFe_2_As_2_, and the inadequacy of a fully localized or fully itinerant description of the 122 family of Fe pnictides.

## Introduction

Soon after the discovery of high temperature superconductivity in Fe pnictides^1^, antiferromagnetic ordering in the form of a spin-density wave has been observed in the parent compounds^2, 3^. The nature of this antiferromagnetism has been highly debated, as demonstrated by the use of antipodal theoretical descriptions, namely, the itinerant and the localized one^2–7^. In the former, magnetism arises from Fermi surface nesting in a similar way to metallic Cr^8^, where this phenomenon leads to spin-density wave ordering caused by a diverging susceptibility at the nesting wavevector. In Fe pnictides, the discovery, by means of angle resolved photoemission spectroscopy, of cylindrical hole and electron pockets satisfying these nesting conditions supported such a picture, together with the metallic ground state, and apparently low electronic correlations^2, 3, 9–11^. However, this weak-coupling scenario could not explain some characteristic properties of Fe pnictides, such as the presence of magnetic moments (*μ*) at high temperature, outside the antiferromagnetic phase, and the persistence of spin excitations in non-magnetically ordered phases^6, 12–20^. These two aspects are more consistently explained in a strong-coupling picture, where strong electronic correlations localize the spins as in Mott-Hubbard-like scenarios^21–23^. However, the metallicity and low *μ* of Fe pnictides conflict with such an extreme strong coupling description.

A formalism which can handle both the itinerant and localized nature of electrons is the dynamical mean field theory (DMFT)^24^. Thanks to fairly recent methodological advances^25, 26^, this formalism can efficiently handle the strongly-correlated metal regime of multi-orbital Hubbard models, such as those relevant for the description of Fe pnictides. An important theoretical prediction from DMFT studies^27–30^ is the phenomenon of *spin-freezing* (SF). In systems with strong Hund’s-coupling, long-lived magnetic moments appear in the metal phase, if the filling and interaction strength place the system in the vicinity of the half-filled Mott insulator. The magnetic moment has been measured in BaFe_2_As_2_^2, 3, 5, 6, 15^, but scant spectroscopic information is available on the temperature and doping effects on *μ*. Moreover, the electron itinerancy, i.e. the dynamics of the electrons, leads to quantum fluctuations, which by time-averaging mask the value of *μ* observed by slow probes (i.e. neutron diffraction, NMR, and muon relaxation measurements^7, 20, 31–42^), making it difficult to extract the “bare” value of *μ*. Fast spectroscopies, probing at the timescale of the electron dynamics (on the order of femtoseconds), are therefore essential to obtain snapshots of the value of *μ*. This is achieved by the use of techniques such as photoelectron, X-ray absorption, and X-Ray emission spectroscopy^14–20, 43^, which indeed produce higher values of *μ* compared to their slower counterparts. Additionally, as explained in refs 17, 44, it is possible to distinguish different aspects of *μ*, the bare *μ* (*μ*_bare_ ∝ 〈*S*_*i*_〉) connected to quantum fluctuations and the correlated *μ* $({\mu }_{corr}\propto \sqrt{\langle {S}_{i}\cdot {S}_{i+1}\rangle })$, which is indicative of dressed quasiparticles (spin excitations). These physical entities represent different aspects of magnetism, have different characteristic time and energy scales, and are probed by different experimental techniques^20^. *μ*_bare_ is detected by local probes such as photoelectron, X-ray absorption, and X-Ray emission spectroscopy^14, 15, 19, 20, 45–47^, whereas *μ*_corr_ is measured by employing inelastic spectroscopies, such as inelastic neutron scattering^6, 48, 49^ and resonant inelastic X-ray scattering^12, 13, 50, 51^.

**Figure 1:**
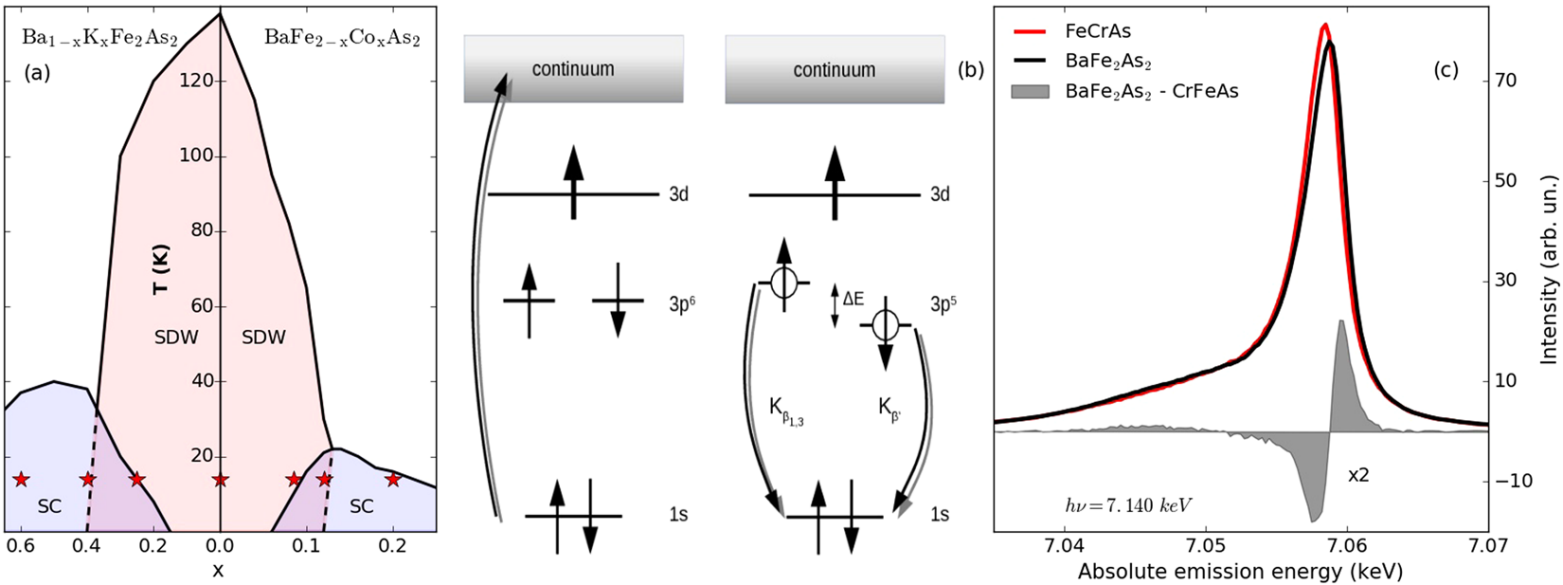
(**a**) Phase diagram of Ba_1−*x*_K_*x*_Fe_2_As_2_ and Ba_1−*x*_K_*x*_Fe_2_As_2_. The red stars depict the doping levels measured. (**b**) Sketch of the XES process. (**c**) Exemplary Fe-K_*β*_ XES for FeCrAs and BaFe_2_As_2_ at 15 K. The former is taken as a reference and the difference spectrum is obtained (see main text) and depicted as gray shadowed curve.

In this paper, we present Fe-K_*β*_ X-ray emission spectroscopy (XES) measurements of *μ*_bare_ in electron- (BaFe_2−*x*_Co_*x*_As_2_) and hole-doped (Ba_1−*x*_K_*x*_Fe_2_As_2_) Fe pnictides. As outlined in Fig. 1(a) by the stars, our study covers a large range of the phase diagram, from underdoped to overdoped for both electron and hole doping. As we will show, at 15 K, in hole-doped compounds, *μ*_bare_ exhibits a weak doping dependence, keeping a value around 1.3 *μ*_*B*_, typical of the parent compound whereas in electron-doped BaFe_2_As_2_, a decrease is observed, with *μ*_bare_ being gradually quenched to 1.1 *μ*_*B*_ (or 85% of the values of the parent) for the most overdoped sample. While increasing the temperature to 300 K enhances *μ*_bare_ in all samples, this effect is more pronounced in hole-doped samples than in electron-doped ones. This shows the inadequacy of a fully itinerant approach to explain the formation of local moments and underlines the importance of Hund’s-coupling and electronic correlations in Fe pnictides.

A much more consistent explanation of the doping and temperature evolution of *μ*_bare_ can be given, with the aid of DMFT calculations, in terms of SF. In BaFe_2_As_2_, the nominal *d* ^6^ occupation and intermediate strength of the electronic correlations imply that the undoped compound is close to the SF crossover regime^30^. Upon hole doping, as the *d*-filling approaches *n*_*d*_ = 5 (half-filling), the effect of the Hund’s-coupling increases, frozen moments appear, and the resulting scattering leads to short quasi-particle life-times and an ill-defined bandstructure. Electron doping, on the other hand, results in a more conventional Fermi-liquid metal, with a well-defined bandstructure and Fermi surface. The electronic screening of *μ*, by a multi-channel Kondo effect^52^, leads to an unusual temperature dependence: *μ* increases with increasing temperature due to a weaker screening effect. Frozen moments with very low Kondo screening temperature appear in the strongly hole-doped region, while electron doping nudges the system towards a more conventional Fermi liquid state with a reduced *μ*. In the spin-freezing crossover regime, the Kondo screening temperature varies strongly with doping and we hence expect a large temperature variation of the local moment.

## Results and Discussions

XES has been established as an extremely sensitive technique in the detection of *μ*_bare_^14–16, 43, 45–47, 51, 53–55^. In this spectroscopy a core-electron from the Fe 1*s* core-shell is excited into the continuum by a photon (in our case *hν* = 7.140 keV), the core-hole is then filled up by a Fe 3 *p* electron together with the emission of a photon (*hν* = 7.040–7.065 keV), as shown by the scheme in Fig. 1(b). The final state, being Fe 3 *p* ^5^, has a wavefunction partly overlapping with the Fe 3 *d* orbitals, which is consequently affected by the spin polarization of the valence band^56, 57^. This gives rise to a main emission line (composed of K_*β*1_ and K_*β*3_) and a satellite peak (K_*β*′_) as shown in Fig. 1(b). The relative intensity of these peaks directly depends on the Fe 3 *d* net spin^14–16, 43, 45–47, 51, 53–55^, and employing a calibration procedure, a quantitative determination of *μ*_bare_ is possible. This method probes the fs timescale^20^ allowing the measurement of *μ*_bare_ ∝ 〈*S*_*i*_〉 and minimizing the problem of electron dynamics decreasing the measured value of the moment. By probing the femtosecond fluctuations of the magnetic moment, this technique gives access to the ultrafast dynamics of the local magnetism. However, it is important to differentiate it from time resolved and pump-probe experiments, which can tune and control the probed time scale.

In Fig. 1(c), we show XES spectra obtained from FeCrAs and BaFe_2_As_2_. The former is employed as a standard material due to *μ*_bare_ = 0 on the Fe sublattice, together with a similar Fe coordination to the samples investigated^58, 59^. BaFe_2_As_2_ has been employed as the high *μ*_bare_ standard, setting it to a value of 1.3 *μ*_*B*_ taken from ref. 15. To determine *μ*_bare_, we employed the integrated absolute difference (IAD) method described in ref. 54. The areas of the spectra are normalized and the difference to the reference spectrum of FeCrAs is calculated. The integration of this difference gives the IAD, which is proportional to *μ*_bare_. To calibrate the absolute energy, we aligned in an additional step all the spectra to the centre of mass as described in ref. 60. We show the difference between the parent and reference compound as the shadowed part of Fig. 1(c). From the integration of this difference spectrum we obtain the IAD.

**Figure 2:**
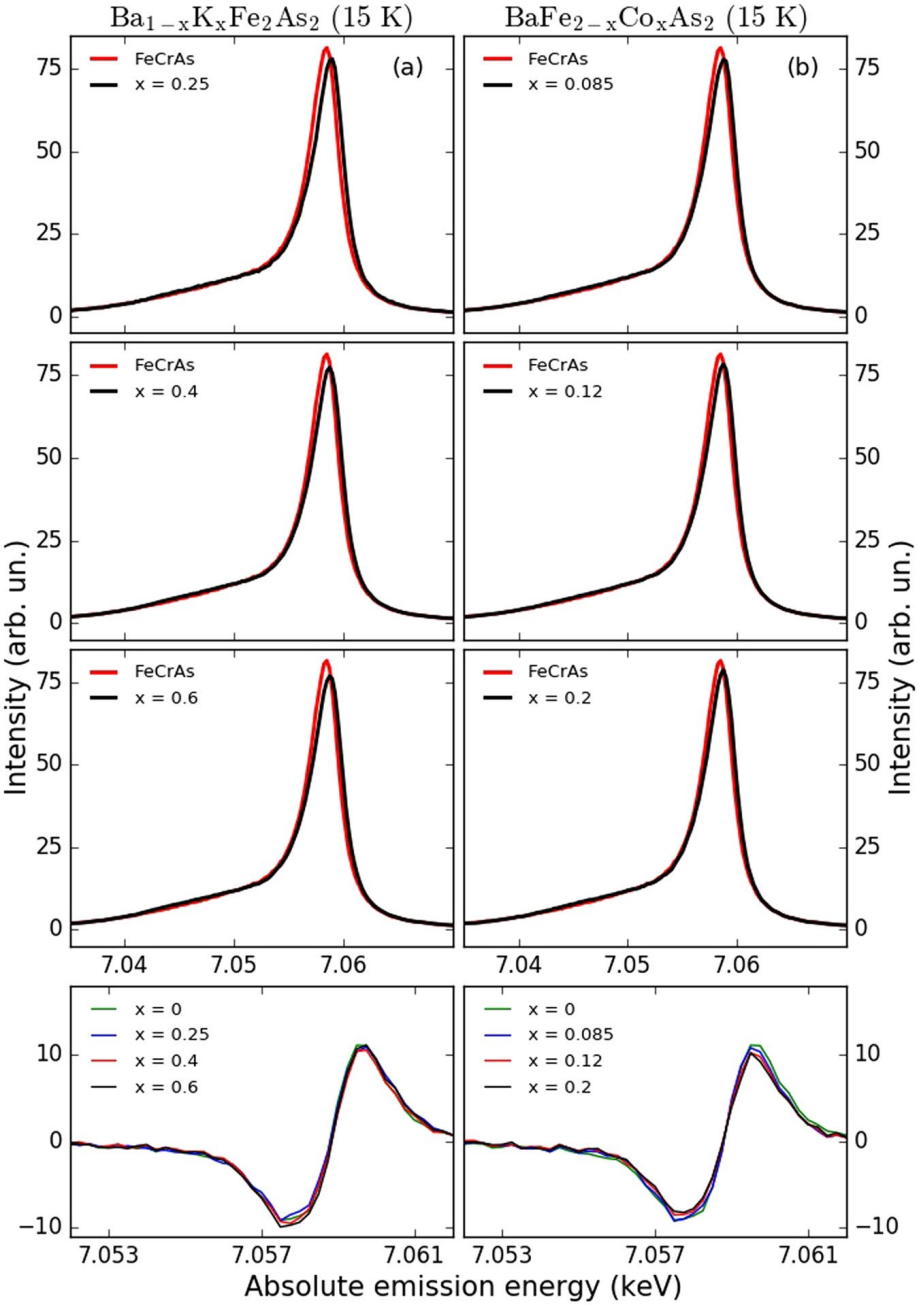
K_*β*_ XES for Ba_1−*x*_K_*x*_Fe_2_As_2_ (**a**) with *x* = 0.25, 0.4, and 0.6 and Ba_1−*x*_K_*x*_Fe_2_As_2_ (**b**) with *x* = 0.085, 0.12, and 0.2 at 15 K. The last row is indicating the relative difference spectra for Ba_1−*x*_K_*x*_Fe_2_As_2_ and Ba_1−*x*_K_*x*_Fe_2_As_2_.

Having calibrated the instrumental response of IAD vs. *μ*_bare_, we now quantify *μ*_bare_ in the doped compounds of BaFe_2_As_2_. In Fig. 2(a), we present the evolution of the XES for hole-doped Ba_1−*x*_K_*x*_Fe_2_As_2_ samples with *x* = 0.25, 0.4, and 0.6 at 15 K. All spectra look very similar with almost no modification detectable. Consequently, the difference spectra shown in the bottom panels of Fig. 2(a) display little change of *μ*_bare_ with hole doping. Moving to the XES spectra of electron-doped Ba_1−*x*_K_*x*_Fe_2_As_2_ (*x* = 0.085, 0.12, and 0.2) depicted in Fig. 2(b), we observe similar spectral features compared to hole-doped BaFe_2_As_2_. However, the IAD analysis shows here a decrease of *μ*_bare_ from 1.3 ± 0.15 *μ*_*B*_ to 1.1 ± 0.15 *μ*_*B*_ with Co doping.

**Figure 3:**
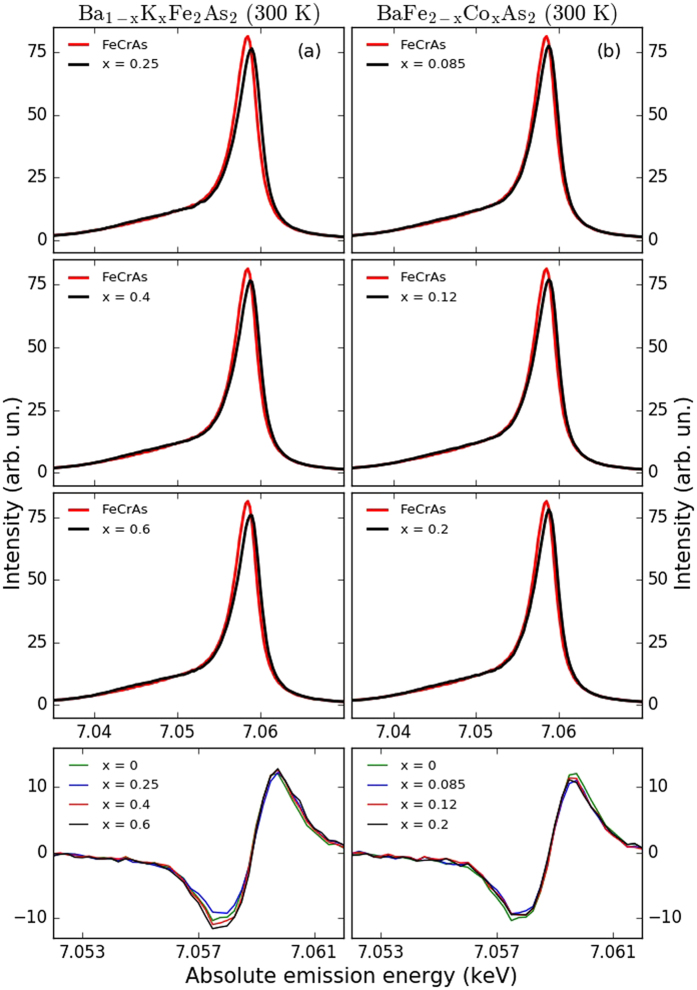
K_*β*_ XES at 300 K for Ba_1−*x*_K_*x*_Fe_2_As_2_ (**a**) with *x* = 0.25, 0.4, and 0.6 and Ba_1−*x*_K_*x*_Fe_2_As_2_ (**b**) with *x* = 0.085, 012, and 0.2 at 300 K. The last row is indicating the relative difference spectra for Ba_1−*x*_K_*x*_Fe_2_As_2_ and Ba_1−*x*_K_*x*_Fe_2_As_2_.

**Figure 4:**
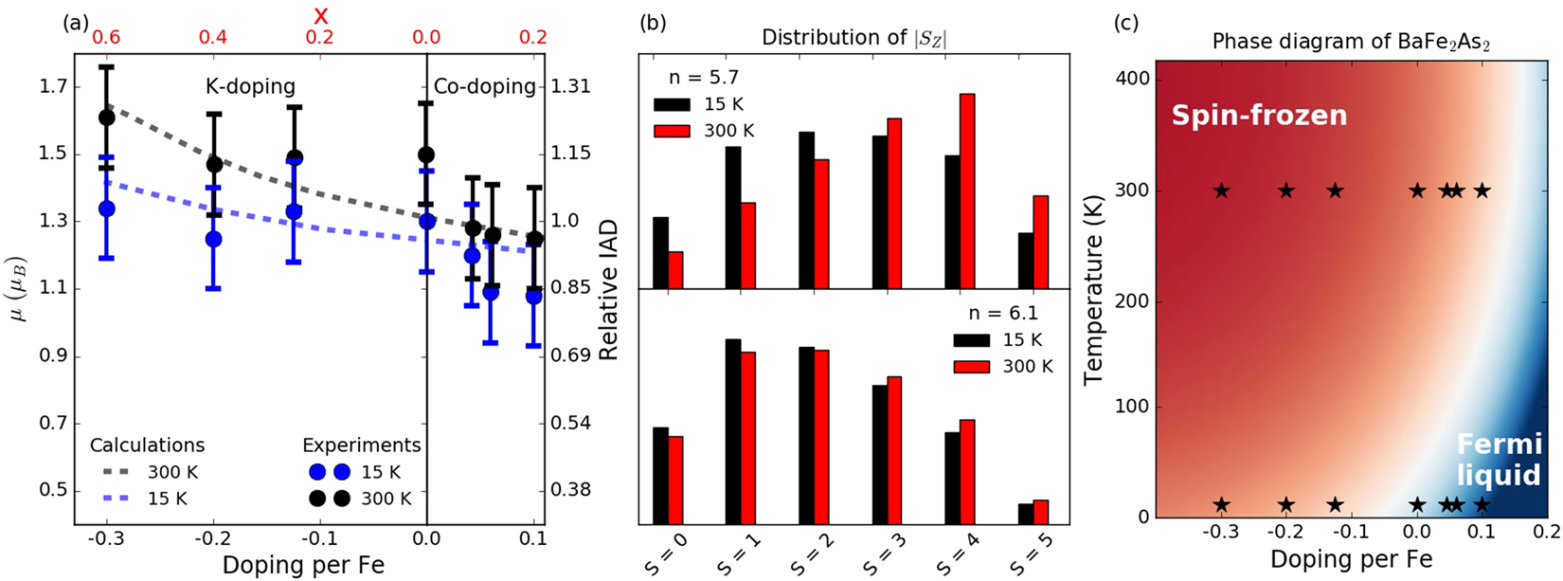
(**a**) Evolution of *μ*_bare_ and relative IAD for Ba_1−*x*_K_*x*_Fe_2_As_2_ and Ba_1−*x*_K_*x*_Fe_2_As_2_. The blue dots with error bars indicate measurements at 15 K, while the black dots with error bars represents *μ*_bare_ at 300 K. The dashed coloured lines are values for *μ* obtained from the DMFT calculations. The relative IAD scale is set to unity for BaFe_2_As_2_ at 15 K. (**b**) Distribution of |*S*_*z*_| values (in units of 1/2) in the thermal ensemble for *n* = 5.7 (top) and *n* = 6.1 (bottom) at 15 and 300 K. (**c**) Sketch of the theoretical phase diagram for Ba_1−*x*_K_*x*_Fe_2_As_2_ and Ba_1−*x*_K_*x*_Fe_2_As_2_ displaying the spin-frozen and Fermi liquid regimes and their evolution with doping and temperature.

We collected additional XES spectra at 300 K and plot them in Fig. 3(a) for Ba_1−*x*_K_*x*_Fe_2_As_2_ (with *x* = 0.25, 0.4, and 0.6) and in Fig. 3(b) for Ba_1−*x*_K_*x*_Fe_2_As_2_ (with *x* = 0.085, 0.12, and 0.2). The spectral shape is basically invariant with temperature, but the XES measurements at 300 K exhibit an *increase* of IAD and consequently an increase of *μ*_bare_ in all samples compared to the respective values at 15 K (Figs 3 and 4(a)).

These measurements are summarized in Fig. 4(a), where we plot the extracted *μ*_bare_ for all dopings at both temperatures. Additionally to *μ*_bare_, we show in Fig. 4(a) on the right-hand side a relative scale of the IAD. In this scale, the IAD of the BaFe_2_As_2_ at 15 K is set to one and the relative change is displayed for all the other compounds. At 15 K, *μ*_bare_ remains approximately 1.3 *μ*_*B*_ in the hole-doped compounds and gradually decreases with doping in electron-doped compounds. This variation is remarkable considering the smaller number of electrons doped by Co-doping compared to the holes injected by K-doping as displayed in the bottom scale of Fig. 4(a). At 0.3 doped holes per Fe no change is observed, whereas doping of just 0.1 electrons per Fe induces a 15% decrease of *μ*_bare_. All the samples display an increase of *μ*_bare_ with temperature, however this increase is surprisingly stronger on the hole-doped samples than in electron-doped ones as shown in Fig. 4(a).

We can partially explain our observations at low temperature by initially considering the fully itinerant limit, where the nesting strength and *μ* are connected, and can be quantified by the Lindhard function, which has been observed to evolve asymmetrically upon doping^61^. The nesting strength decreases linearly with the injection of electrons, but remains constant up to *x* = 0.4 for hole doping where it starts to decrease for even larger doping^61^. This could account for the decrease of *μ*_bare_ upon electron doping and partially explain the almost constant *μ*_bare_ for weak hole doping, but it clearly fails at higher hole doping concentrations.

Moreover, the lack of magnetic ordering, and the observation of a paramagnetic state with an increased *μ*_bare_ at 300 K, demonstrates that a Fermi surface nesting scenario completely fails to describe the evolution of *μ* at high temperature. Theoretical work suggested the importance of the Hund’s coupling interaction and the need to combine local and itinerant physics to explain the magnetism of Fe pnictides^62–64^. The effect of doping has also been studied from both a theoretical and experimental standpoint, with the conclusion that doping does not only affect the carrier density and the chemical potential but also that the disorder induced by doping has to be taken into account, which can in principle account for the enhanced scattering rate upon doping^65, 66^.

Neutron scattering measurements of *μ*_corr_ show a good agreement with our findings on electron-doped samples^49^, but a decrease is observed on hole-doped samples^48^. A difference in the evolution of magnetism upon hole doping has also been reported in INS and RIXS measurements of the spin excitations in Ba_1−*x*_K_*x*_Fe_2_As_2_^12, 48^. Both techniques detected consistently a decrease of the bandwidth of the spin excitations, but different results are observed in the intensity. INS detected a decrease of total intensity^48^, whereas RIXS experiments showed a constancy in the intensity of the spin excitations^12^. This dichotomy may arise from the different region of the BZ zone probed by the two techniques^6^, with INS having high sensitivity close to the antiferromagnetic ordering vector and RIXS measuring close to the Γ point. Generally this considerations make our data in agreement with RIXS experiments close to the Γ point.

When compared with our XES measurements, it is important to remember that *μ*_*bare*_ from XES, *μ*_*corr*_ probed by INS, and the spectral weight in RIXS have different correlation lengths. Our XES measurements probe the magnetic moment localized on a single Fe atom, whereas INS and RIXS can integrate along the momentum and energy domain obtaining *μ*_*corr*_ which is sensitive to to collective magnetic excitations. Thus in summary, they probe different aspects of the magnetism.

To aid the interpretation of the experimental measurements, we performed DMFT simulations of a five-orbital Hubbard model with a semi-circular density of states (DOS) of bandwidth 4 eV, which corresponds to the *d*-electron bandwidth of Ba_1−*x*_K_*x*_Fe_2_As_2_ in the local density approximation^30^. The Coulomb interaction matrix was taken from ref. 30, but re-scaled in such a way that the SF crossover in the model with the simplified DOS occurs near *d*-electron filling *n*_*d*_ = 6 at temperature *T* = 100 K. (The fluctuating local moments at the border of the spin-frozen regime lead to a characteristic $\sqrt{\omega }$ frequency dependence of the self-energy^27^, which can be used to identify this crossover regime.) We solved the DMFT equations using the hybridization-expansion approach^25^, restricting the solution to paramagnetic metal states. The hybridization-expansion method gives direct access to the fluctuating Fe-3*d* states, and allows to calculate the instantaneous *μ* (here estimated as $\mu \approx \sqrt{\langle {S}_{z}\cdot {S}_{z}\rangle }$) in the relevant temperature and doping regime.

The calculations yield magnetic moments between 1.25 and 1.65 *μ*_*B*_, in good agreement with the experimental results. We show the simulation results for temperatures *T* = 15 K and 300 K as dashed lines in Fig. 4(a). They display an increase of *μ* with hole doping and a decrease with electron doping in qualitatively good agreement with the experiments. The doping evolution can be ascribed to a change in the Fe-3 *d* filling, which affects the distribution of atomic states in the thermal ensemble. In particular, electron doping (hole doping) moves the system further away from (closer to) filling *n*_*d*_ = 5, which is needed to realize the maximum spin state in a localized picture. (In the experiments, the formal occupation is 3 *d* ^6.1^ and 3 *d* ^5.7^ at the highest dopings.) Most interestingly, our calculations also predict an increase of *μ* with increasing temperature, an effect which is particularly pronounced on the hole-doped side. Within the SF picture, this arises from a weaker Kondo screening of the local moments at high temperature. In this context, future experimental studies at intermediate temperatures might elucidate whether the magnetic moment increases continuously or if some discontinuous temperature development occurs. It is also instructive to look at the distribution of |*S*_*z*_| values in the thermal ensemble, which is plotted in panel (b) of Fig. 4. Especially on the hole-doped side, these histograms provide clear evidence for a weight shift towards high-spin states and reduced spin fluctuations at the higher temperature.

By correctly reproducing the experimentally observed stronger increase of *μ*_bare_ with temperature in hole-doped samples, our DMFT calculations confirm that this behaviour is a signature of a crossover into a spin-frozen state. Figure 3(c) illustrates the consequences of the SF crossover on the nature of the metallic phase together with the respective position of the measured samples in the phase diagram (black stars). Hole doping shifts the Fe configuration towards half-filling, and the strong scattering from frozen moments wipes out the bandstructure and invalidates Fermi surface nesting arguments. On the other hand, electron doping leads the system away from the SF crossover region into a more conventional correlated metal regime, indicated by the blue region, where Fermi surface nesting arguments are applicable. This picture is consistent with recent optical measurements showing a non-Fermi liquid response for hole-doped BaFe_2_As_2_ and Fermi liquid behaviour for electron-doped BaFe_2_As_2_^67^.

The difference in slopes observed between calculations and experiments in the electron-doped region and at low temperature may be explained as a consequence of competition between the Fermi surface nesting and the SF. As it is clear from Fig. 4(c), this is the only region of the explored phase diagram where there is a Fermi liquid phase. This suggests that Fermi surface nesting prevails over SF, so that the decrease of *μ*_bare_ arises mainly from a worsened nesting. This effect is not captured by DMFT calculations with a semi-circular DOS and is difficult to implement in such a theoretical framework. In this case a theoretical framework accounting for the detailed band structure of the system should be used instead of a simplified semi-circular DOS. The situation is opposite on the hole-doped side where Hund’s-coupling and SF effects dominate nesting and *μ*_bare_ is more strongly affected by local physics. Despite the difficulty of our model to simulate the low temperature electron-doped region, it is remarkable that such a model based on few parameters can cover the evolution of the magnetic moment in a wide portion of the phase diagram as a function of both doping and temperature.

Another consideration concerns the *c* lattice parameter, which has been connected to the magnitude of the magnetic moment^6, 68^. Specifically, the *c* lattice parameter has been observed to shrink in Co-doped samples and to increase in K-doped samples^69, 70^, implying an increase of hopping with Co doping leading to a Fermi liquid phase, and a decrease of hopping in the K-doped phase driving the system into a more correlated phase, i.e. the SF region. This connection between *c* lattice parameter and electronic correlations is in agreement with our interpretation of the evolution of *μ*_bare_ within the phase diagram, since the interaction effects are increased in hole-doped samples and decreased on the electron-doped side of the phase diagram (especially at low temperature).

## Conclusions

In summary, we have measured *μ*_bare_ in hole- and electron-doped BaFe_2_As_2_ across the phase diagram. At 15 K, we found *μ*_bare_ to be weakly dependent on hole doping, but to clearly decrease upon electron doping, in agreement with a crossover between a SF phase and a correlated metal phase with well-defined Fermi surface. Our work demonstrates the importance of Hund’s coupling in the description of Fe pnictides. The asymmetrical increase of *μ*_bare_ at 300 K results from a competition between electronic screening and Hund’s-coupling induced local moment formation. The qualitative agreement between the doping and temperature dependence observed in both theory and experiment indicates that a SF occurs in hole-doped BaFe_2_As_2_, and that both Hund’s-coupling and nesting effects are essential for understanding the unconventional metal state of Fe pnictides.

## Methods

Single crystals of BaFe_2_As_2_, Ba_1−*x*_K_*x*_Fe_2_As_2_, and Ba_1−*x*_K_*x*_Fe_2_As_2_ have been grown by the flux method as described in refs 71, 72. We performed XES experiments at BL11XU of SPring-8, Hyogo, Japan. The incoming beam was monochromatized by a Si(111) double-crystal and a Si(400) secondary channel-cut crystal. The energy was calibrated by measuring X-ray absorption of an Fe foil and set to 7.140 keV with *π* polarization. We employed three spherical diced Ge(620) analyzers and a detector in Rowland geometry at ca 2 m distance. The total combined resolution was about 400 meV estimated from FWHM of the elastic line. We scanned the absolute emission energy between 7.02 keV and 7.08 keV and normalized the intensity by the incident flux monitored by an ionization chamber. We carried out measurements at both 15 and 300 K.
